# New Appliances and Things Medical

**Published:** 1899-01-21

**Authors:** 


### NEW APPLIANCES AND THINGS MEDICAL.

[We shall be glad to receive, at our Office, 28 & 29, Southampton Street, Strand, London, W.C., from the manufacturers, specimens of all new preparations and appliances which may be brought out from time to time.]

### THE "BUSY MOTHER INFANTS' FOOD."

(BLEASDALE & CO., LIMITED, 23, COLLIERGATE, YORK.)

THE Busy Mother Infants' Food is said to be a perfect food for infants and invalids, containing in an easily digested form all the necessary elements for the building up of healthy muscle, flesh, and bone. Examination of a sample showed it to consist of a partially cooked cereal flour, containing a good proportion of albumenoids or flesh-forming substances, and more than the usual proportion of soluble carbohydrates. It makes a very palatable, nutritious, and easily digested food for children and invalids, and for infants when their digestive organs have reached the stage of development necessary for the digestion of amylaceous substances.

### THE "GLEBE" PURE CANE SUGARS.

(GLEBE SUGAR REFINING COMPANY, GREENOCK, AND ST. GEORGE'S HOUSE, EASTCHEAP, E.C.)

THE Glebe Sugar Refining Company have submitted samples of their sugar, which is guaranteed to be prepared from the cane and not from the beet. Examination of the samples of cube, castor, and granulated sugar shows that there is every indication of this statement being correct. The samples were practically pure sugar. They were free from all mineral constituents or ash, and only contained an infinitesimal amount of invert sugar. These results indicate a high degree of refining in order to produce sugars answering to these characters. If only on the ground of supporting our own colonies, the use of cane sugar, as distinct from beet sugar, is worth encouragement.

